# An overabundance of phase 0 introns immediately after the start codon in eukaryotic genes

**DOI:** 10.1186/1471-2164-7-256

**Published:** 2006-10-11

**Authors:** Henrik Nielsen, Rasmus Wernersson

**Affiliations:** 1 Center for Biological Sequence Analysis, Technical University of Denmark, Building 208, 2800 Lyngby, Denmark

## Abstract

**Background:**

A knowledge of the positions of introns in eukaryotic genes is important for understanding the evolution of introns. Despite this, there has been relatively little focus on the distribution of intron positions in genes.

**Results:**

In proteins with signal peptides, there is an overabundance of phase 1 introns around the region of the signal peptide cleavage site. This has been described before. But in proteins without signal peptides, a novel phenomenon is observed: There is a sharp peak of phase 0 intron positions immediately following the start codon, *i.e*. between codons 1 and 2. This effect is seen in a wide range of eukaryotes: Vertebrates, arthropods, fungi, and flowering plants. Proteins carrying this start codon intron are found to comprise a special class of relatively short, lysine-rich and conserved proteins with an overrepresentation of ribosomal proteins. In addition, there is a peak of phase 0 introns at position 5 in *Drosophila *genes with signal peptides, predominantly representing cuticle proteins.

**Conclusion:**

There is an overabundance of phase 0 introns immediately after the start codon in eukaryotic genes, which has been described before only for human ribosomal proteins. We give a detailed description of these start codon introns and the proteins that contain them.

## Background

Ever since eukaryotic genes were discovered to be interrupted by introns, there has been a heated debate about the origin and evolution of introns. The "introns-early" school believes that introns were present in the last universal common ancestor of pro- and eukaryotes, and that intron loss is responsible for the lack of introns observed in bacteria. The "introns-late" school, on the other hand, believes that introns have appeared during the evolution of the eukaryotic lineage, and that intron gain is a frequent event in the evolution leading to the gene structures we see today. This debate continues to generate a huge amount of literature; for a recent review, see Rogozin *et al*. [1].

On this background, it is surprising that the question of intron position distribution in eukaryotic genes has received relatively little attention. As an exception to this, it has been observed that introns are not uniformly distributed over the entire gene, but tend to be more abundant close to the 5' end. This locational bias is especially seen for genes with only a single intron. Sakurai *et al*. [2] found the 5' bias for genes with a single intron in 6 out of 7 genomes studied (it was absent in *Arabidopsis thaliana*). In the unicellular organisms *Saccharomyces cerevisiae *and *Plasmodium falciparum*, which have relatively intron-poor genomes, there was a marked 5' bias for all introns. Mourier and Jeffares [3] found that the 5' bias was only seen in intron-poor genomes of unicellular organisms. Interestingly, they found no 5' bias in *Plasmodium *where Sakurai *et al*. [2] had seen it. Recently, Lin and Zhang [4] investigated 21 complete eukaryotic genomes and reported that 5' bias was found in all of them, including both uni- and multicellular organisms. They used a different way of testing this than the other two groups: instead of normalizing all intron positions to a number of bins and adding them up before doing statistical tests, they treated each gene as an independent test and recorded its intron positions as 5' biased, 3' biased, or equally distributed. It is hypothesized that the origin of the 5' bias is related to the mechanism of intron loss: a spliced mRNA can be converted to an intron-less cDNA by reverse transcription, and if the cDNA then recombines with the gene, one or more introns are lost. Since the reverse transcriptase begins from the 3' end of the mRNA, incomplete cDNAs predominantly represent the 3' end of the gene, and therefore, intron loss preferentially occurs in the 3' end [2,3].

Also intron gain seems to occur preferentially in the 3' end, Sverdlov *et al*. [5] reported. They found that phylogenetically old introns (with positions conserved between distant phylogenetic lineages) showed an excess in the 5' end, while new introns in intron-rich genomes were found preferentially in the 3' end. The 5' end of an intron is referred to as the *donor *site, and the 3' end as the *acceptor *site. The position of the donor and acceptor sites relative to the reading frame is referred to as the *phase *of the intron: a phase 0 intron is positioned between two codons, while a phase 1 intron disrupts a codon after the first position and a phase 2 intron after the second position. In most coding regions, phase 0 introns are the most common, followed by phase 1 introns and then phase 2 introns as the least common [6,7]. In proteins with secretory signal peptides, however, phase 1 introns are the most common [8].

Introns are recognised by the spliceosome, a complex of several small ribonucleoprotein particles (snRNPs) [9]. There is an almost completely conserved consensus sequence for the donor and acceptor sites, the two first positions in the intron being "gt" and the last two positions "ag". Exceptions to this rule exist, but they are rare. Burset *et al*. [10] did a comprehensive analysis of EST-supported canonical and non-canonical splice sites and reported that 1.29% of the introns, after correcting errors, had sequences other than "gt...ag." The majority of these (approximately half) were "gc...ag," and the other patterns each comprised less than 0.05%.

It has been found that introns with the non-canonical sequence "at...ac" comprise a special group recognised by its own spliceosome, where the rare U11 and U12 snRNPs have replaced the standard U1 and U2 particles [11].

There is also some weaker sequence conservation on the exon side of donor and acceptor sites. The consensus sequence for the exon-exon junction is "ag|g" where the "|" denotes the position of the intron [12]. This has been described as the "proto-splice site" as it is assumed that new introns are predominantly inserted into an "ag|g" site [13]. Coghlan and Wolfe [14] indeed found that recently gained introns (as inferred from phylogenetic analyses) had a stronger "ag|g" consensus than older introns. However, the "introns-early" school, who tends to be skeptical of the notion of intron gain, has found that the occurrence of "proto-splice sites" does not agree with the distribution of intron phases found in extant organisms [15]. The existence of a nucleotide consensus flanking existing introns is, of course, no guarantee that the sites are remnants of original proto-splice sites; they might also have evolved convergently after the introns appeared in order to adapt to the splicing machinery. Sverdlov *et al*. [16] addressed this question by examining the context of introns inserted at amino acids that were totally conserved between eight diverse eukaryotic genomes. By considering only nucleotides that could not be changed without changing the amino acid, they arrived at a splice site sequence context that had not been modified by directional selection, and it turned out to have the same consensus as that of all splice sites. The conclusion is that either introns have been inserted preferentially into proto-splice sites, or they have been inserted at random but preferentially fixed if the sequence context was a proto-splice site.

Another question is whether shared intron positions reflects evolutionary conservation or parallel gain of new introns. Qiu *et al*. [17], using a Bayesian modeling of intron evolution, found that most introns shared between distantly related species are results of parallel gains. This is in contrast to two more recent papers. Sverdlov *et al*. [18] constructed a dynamic model of intron insertion by using a weight matrix for the proto-splice sites and inserting introns with a probability proportional to the weight matrix score. Their simulated results suggest that only a small fraction (5–10%) of shared intron positions in distantly related species are due to parallel gains. Nguyen *et al*. [19] used a maximum likelihood estimator where the number of target sites (potential intron positions) was treated as an parameter to be estimated, and their results suggest that parallel gains account for ≈18.5% of shared intron positions. When comparing the genes for cytoplasmic ribosomal proteins to those for mitochondrial ribosomal proteins, however, the same group found that all shared intron positions between these two groups resulted from parallel gains [20]; a result consistent with the introns-late view that the bacterial ancestor of mitochondria did not have introns. 

It is a matter of debate whether intron position correlates with protein structure. If the "introns-early" theory, also called the "exon theory of genes" is correct, proteins should have evolved by assembly of small autonomous modules or structural domains, and one should expect that exons in proteins will correspond to boundaries of such modules. Stoltzfus *et al*. [21] tested this and found no correlation between intron position and protein structure. On the other hand, de Souza *et al*. [22] found that phase 0 introns were indeed found at boundaries of compact protein modules, and Fedorov *et al*. [23] reported that this was especially true for phylogenetically old proteins (common to pro- and eukaryotes). Recently, Whamond and Thornton [24] analysed intron positions in relation to amino acids and protein secondary structure. They found that the distribution of intron positions in the three structural classes helix, sheet, and coil was different from the background distribution, but this bias could be largely explained by the nucleotide preferences surrounding the introns (the proto-splice sites).

Tordai and Patthy [25] investigated the distribution of introns in human genes with and without signal peptides, and found a significant excess of phase 1 introns in the vicinity of the signal peptide cleavage site. The reason for this is hypothesized to be that proto-splice sites of phase 1 correspond to glycine codons ("ggn"), and positions -1, -3, -4 and -5 relative to the cleavage site are significantly enriched in glycine. It also correlates well with the fact that extracellular proteins often have evolutionary modules or domains that are bounded in both ends by phase 1 introns [26].

Recently, Vibranovski *et al*. [8] (from the "introns-early" side) reinvestigated this phenomenon and reported that all "g|g" intron contexts, not only "ag|g" proto-splice sites, were enriched in the region of the signal peptide cleavage site, and that the entire sequence of proteins with signal peptides were enriched in phase 1 introns. On this background, they claim that exon shuffling, rather than intron insertion into proto-splice sites, must be the explanation for the phase 1 signal peptide peak.

## Results

### Intron length distribution

During splicing, the intron assumes a lariat structure, where the 5' end is covalently attached to a branch point a short distance upstream of the 3' end [9]. The intron must have a certain minimum length for this lariat formation to be possible, but exactly how short an intron can be is a matter of some debate. Goodall and Filipowicz [27], using deletion mutants, found that the minimum functional intron length in plants (both monocots and dicots) was between 70 and 73 nt. They noted that this length requirement is similar to that seen in vertebrates, but significantly greater than that in fungi and insects.

Introns shorter than this occur in GenBank. The shortest of them are probably annotation errors, but where should we set the cutoff? In order to find a non-arbitrary lower length threshold for introns, the intron length distribution was calculated on the GenBank data – vertebrates, arthropods, fungi, and flowering plants (*Magnoliophyta*).

In the length distribution dataset, introns without the canonical "gt...ag" sequence were not weeded out. In all organism groups, the second most abundant splice site sequence was "gc...ag", comprising 0.58–1.14% of the introns (higher in fungi than in the three other groups). This is in agreement with earlier observations [10]. Intron length distribution was calculated for introns with non-consensus splice sites separately, and it was found that many of them (up to 22.6% in vertebrates) were less than 5 nucleotides in length. In fact, these gaps in the coding sequence probably do not represent introns, but programmed translational frameshifts [28].

**Figure 1:**
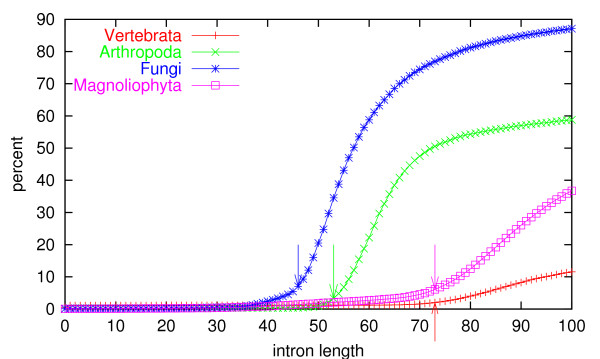
Cumulated length distribution of introns from the four organism groups. The arrows show the chosen values for minimum length cutoff.

In Figure 1, the cumulative distribution of intron lengths in the four organism groups is shown. It is apparent that the length distributions are very different for the four groups. Introns shorter than 100 nt accounted for 86.9% of the fungal introns, but only 11.3% of the vertebrate introns. The non-cumulated distributions of intron lengths are shown in Supplementary Figure S1 [see Additional file 1].

The curves in Figure 1 all have a linear domain. We defined a minimum length cutoff by drawing a line through the linear domain and extending it to the x-axis. This yielded the following thresholds: vertebrates: 73 nt; arthropods: 53 nt; fungi: 46 nt; and plants: 73 nt. These values are in agreement with those of Goodall and Filipowicz [27].

### Intron position statistics

**Figure 2:**
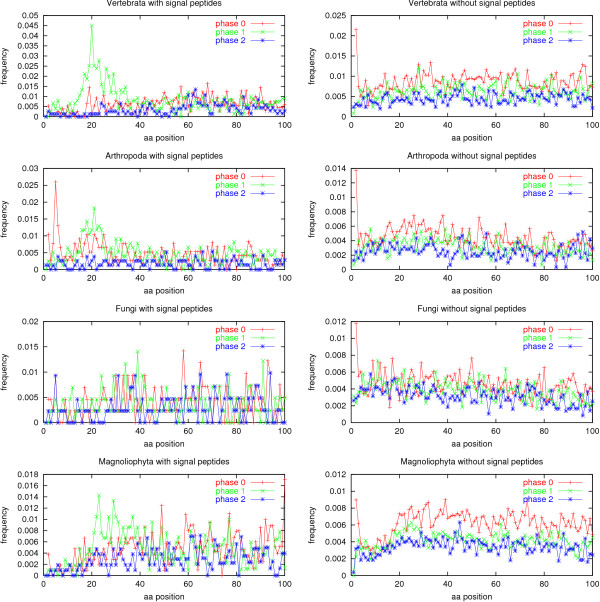
Intron positions in the 100 most N-terminal amino acids of the coding sequence of eukaryotic genes. Genes are divided into the systematic groups vertebrates, arthropods, fungi, and flowering plants (*Magnoliophyta*). The data sets are homology reduced. Left: genes predicted to code for a protein with a signal peptide; right: genes predicted not to carry a signal peptide. For phase 0 introns the position refers to the amino acid *after *the intron.

The distributions of intron positions within the first 100 amino acid positions of eukaryotic genes from the GenBank sets are shown in Figure 2. In the plots of genes without signal peptides (the right half of the figure), it can be seen that phase 0 introns are more frequent than phase 1 introns which are again more frequent than phase 2 introns, in agreement with what has been described before [6,7]. The excess of phase 0 introns is particularly pronounced in plant genes.

For proteins with signal peptides (the left half of the figure), there is an excess of phase 1 introns in a region around 20 for the two animal groups, and between 20 and 40 in plants. This has been described as corresponding to the positions of the signal peptide cleavage sites [25,8], cf. the Background section. The phase 1 excess cannot be seen for the fungal sequences, where proteins with signal peptides and introns are simply too rare to make this type of statistics.

In proteins without signal peptides (the right half of the figure), another phenomenon is seen: a sharp peak of phase 0 introns at position 2, *i.e*. between the start codon and codon 2. This is what we refer to as *the start codon introns*. Note that there are no values for phase 0 introns at position 1 – this would be introns occurring immediately *before *the start codon, and those are not visible in the GenBank coding sequence ("CDS") features the data are derived from (see Methods).

The peak of start codon introns is very high in vertebrates, arthropods and fungi. In plants, it is not higher than the peaks for phase 0 introns between positions 20 and 40, but it is still conspicuous against the relatively intron-poor region between positions 3 and 20.

Note that introns disrupting the start codon, *i.e*. phase 1 and phase 2 introns in position 1, are relatively rare, especially in plants.

**Figure 3:**
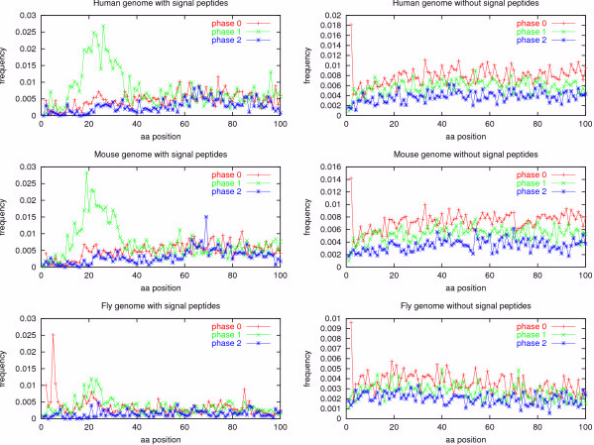
Intron positions in the 100 most N-terminal amino acids of the coding sequence of genes in the *Homo sapiens*, *Mus musculus*, and *Drosophila melanogaster *genomes. The data sets are not homology reduced.

In the genome data sets, both the phase 1 peak for proteins with signal peptides and the start codon peak for proteins without signal peptides are clearly visible (see Figure 3). The intron position distributions for the human genome and the mouse genome look very similar to the vertebrate GenBank set, and the *Drosophila *genome shows the same pattern as the arthropod GenBank set.

Start codon introns have been briefly mentioned in the literature before, but only for human ribosomal proteins. In a paper about the human ribosomal protein genes, Yoshihama *et al*. [29] remarked: "Interestingly, the ATG was always located near the splice sites of the first intron and, in 20 cases, was exactly at the 3' end of the first exon" (the total number of genes analysed was 73).

The phenomenon is not limited to ribosomal proteins, however. To address this question, we made plots of the genome data sets without signal peptides with ribosomal proteins removed (see Methods for details). These plots are shown in Supplementary Figure S2 [see Additional file 1]. In the plots, there is only a slight lowering of the start codon peak; the relative frequency being 0.0160 for human, 0.0122 for mouse, and 0.0083 for fly (compare these values with Figure 3); still well outside the range of frequencies for other positions.

In arthropod genes with signal peptides, a peak of phase 0 introns is seen at position 5 (Figure 2). We checked the GenBank annotation of these 20 proteins and found that 17 of them were from the genome sequence of *Drosophila melanogaster*. Indeed, the same peak can be seen in the non-homology reduced *Drosophila *genome (see Figure 3), with the peak containing 88 proteins. A curious observation is that 18 of the 20 introns and 80 of the 88 introns (90–91%) are the only introns in their respective genes; for comparison, only 44.9% of all the *D. melanogaster *genes with signal peptides have exactly one intron. The functional annotation of the 88 peak proteins reveals a striking fact: 38 of them (43%) are structural constituents of cuticle (adult, larval or pupal). 46 have functional annotation missing or "unknown," and only 4 have an annotation of something other than cuticle. For comparison, only 2.8% of all the *D. melanogaster *genes with signal peptides are annotated with "structural constituent ... cuticle." 

In arthropods, fungi and plants without signal peptides, a slight downward slope can be seen in the intron frequency. We tested whether this is a real 5' bias by computing the correlation coefficient between position and relative frequency for positions 20 through 100. Fungi and plants have significant (*p *< 1%, Pearson test) downward slopes for all three phases, arthropods only for phase 0 and phase 1. The vertebrate set shows no slope.

**Figure 4:**
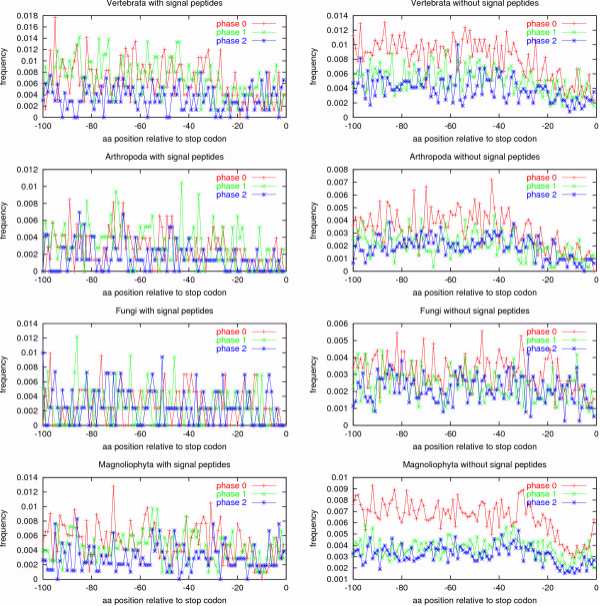
Intron positions in the 100 most C-terminal amino acids of the coding sequence of eukaryotic genes. The data sets are the same as in Figure 2.

If the proteins are aligned by the stop codon instead of the start codon, the picture shown in Figure 4 emerges. There are no stop codon peaks, except for a weak phase 0 peak before the last codon in plants without signal peptides. For proteins with signal peptides, no special features can be seen. Observe that it is only in proteins without signal peptides that phase 0 introns are the most abundant. For proteins without signal peptides, a slight reduction in the intron frequency for all three phases can be seen in the last approximately 20 positions (for fungi only approximately 5).

### Proteins with start codon introns

In order to characterize the start codon introns, they were compared to a background set. Both the start codon introns and the background set were derived from proteins without signal peptides. To eliminate possible bias from the fact that start codon introns are always phase 0 and always the first intron in the coding sequence (note: this does not necessarily mean the first intron in the gene, since there can be introns in the 5' untranslated region (UTR)), the background set was limited to phase 0 introns that were the first in the coding sequence.

A comparison of intron lengths was inconclusive. For vertebrates, start codon introns are slightly shorter than the background set, but for fungi they are slightly longer. For arthropods and plants, there are no significant differences [see Supplementary Table S1 from Additional file 1].

When examining the nucleotide distribution, significant differences are found for vertebrates, fungi, and plants (*p *< 10^-4^, χ^2^-test, *df *= 3), but not for arthropods [see Supplementary Table S1 from Additional file 1]. When using nucleotide pair frequencies, significant differences are found for all four groups (*p *< 10^-3^, χ^2^-test, *df *= 15) [see Supplementary Table S1 from Additional file 1]. However, the results show no clear trends – there are no dinucleotide preferences that are the same in all four organism groups.

The sets of proteins without signal peptides carrying a start codon intron were also subjected to further analysis, and here, some interesting differences were found. Again, both the positive and the negative set consisted of proteins without signal peptides. The first thing we noticed was that proteins with start codon introns are on average shorter than other proteins, see Table 1. The differences are significant on the 5% level for vertebrates and fungi, and better than the 0.1% level for arthropods and plants (Welch two sample t-test).

**Table 1:** Proteins with and without start codon introns

	Vertebrata		Arthropoda		Fungi		Magnoliophyta	
	sci	no sci	sci	no sci	sci	no sci	sci	no sci
length	253.7	333.7	271.3	419.2	289.1	403.7	152.9	211.2

A	6.84%	7.04%	7.56%	7.22%	7.54%	7.74%	8.23%	8.46%
C	1.76%	2.12%	1.36%	**1.91%**	1.08%	1.21%	2.03%	1.94%
D	5.15%	4.86%	5.52%	5.31%	5.37%	5.55%	4.68%	**5.35%**
E	6.69%	7.08%	6.78%	6.61%	**7.28%**	6.57%	6.67%	6.44%
F	3.63%	3.79%	3.48%	3.82%	3.93%	3.72%	3.55%	3.39%
G	**6.85%**	6.26%	5.90%	5.55%	5.89%	**6.46%**	7.90%	7.56%
H	2.49%	2.59%	2.36%	2.60%	2.08%	2.45%	2.47%	2.53%
I	**4.98%**	4.42%	5.18%	5.18%	5.30%	5.10%	4.61%	4.37%
K	**7.94%**	5.95%	**8.08%**	5.89%	**6.77%**	5.53%	**6.52%**	5.20%
L	9.32%	**10.26%**	9.89%	9.88%	9.03%	9.30%	8.61%	8.83%
M	2.40%	2.35%	2.62%	2.51%	1.94%	2.19%	2.60%	2.54%
N	3.45%	3.52%	4.63%	4.59%	**4.57%**	3.87%	3.31%	3.45%
P	5.40%	**5.94%**	4.20%	**5.02%**	5.66%	5.96%	5.10%	5.51%
Q	4.42%	4.75%	4.57%	4.81%	4.32%	3.90%	3.64%	3.52%
R	6.20%	5.77%	**6.43%**	5.84%	5.71%	5.90%	**8.09%**	7.16%
S	6.75%	**7.99%**	6.20%	**7.76%**	8.13%	8.54%	7.16%	**8.54%**
T	5.13%	5.15%	4.98%	5.37%	5.46%	5.71%	4.85%	4.94%
V	**7.01%**	6.20%	6.43%	6.09%	6.08%	6.12%	6.22%	6.58%
W	0.87%	1.27%	0.90%	1.06%	0.97%	1.40%	1.27%	1.41%
Y	2.74%	2.68%	2.92%	3.00%	2.88%	2.78%	2.50%	2.28%

Average length (in amino acids) and amino acid frequencies for proteins without signal peptides, divided into proteins with ("sci") and without ("no sci") start codon introns. Where the difference for a particular amino acid is greater than 0.5%, the higher percentage is shown in boldface.

The amino acid distributions were also calculated, and it was found that there are significant differences between proteins with and without start codon introns for all organism groups (*p *< 10^-15^, χ^2^-test, *df *= 19). The amino acid frequencies are shown in Table 1. The most conspicuous differences, seen in all four organism groups, are that lysine (K) is more abundant in proteins with start codon introns than in other proteins, while serine (S) is less abundant.

In order to assess the conservation of start codon intron proteins, human genome proteins involved in a reciprocal best hit (RBH – see Methods for definition) to mouse or fly were compared. While human proteins with a start codon intron on average have 91.5% identities in the global alignments with mouse, the other proteins have only 84.8% (Welch two sample t-test, *p *< 10^-5^). For the alignments of human versus *Drosophila*, the proteins with start codon introns had 60.9% identities, while the others had 40.2% (two sample t-test, *p *< 10^-15^). Only RBHs where the human gene was predicted not to contain a signal peptide were included in these calculations.

We also tested whether start codon intron positions are more conserved than other intron positions. All intron positions were mapped onto the global alignments, and in positions where the human protein had an intron it was tested if the mouse or fly protein also had an intron. In the comparison of human versus fly, there are 76 RBHs with a human start codon intron. 45 had a corresponding fly phase 0 intron, and 43 of these (56.6%) were also in a start codon position. For comparison, only 2164 of the 16435 (13.2%) human phase 0 introns in other positions are conserved in fly. This difference is highly significant (*p *< 10^-15^, χ^2^-test, *df *= 1). In the comparison between human and mouse, where the overall conservation of phase 0 intron positions is as high as 95.0%, no significant difference is seen.

When looking at the functional annotations, a curious observation was made: in the sets of proteins with start codon introns, ribosomal proteins are overrepresented. This, of course, corresponds well with the fact that ribosomal proteins was the first place where start codon introns were observed. In the vertebrates, there are 16 ribosomal proteins out of the 55 start codon intron proteins, corresponding to 29%, while the background frequency in the non-start codon intron proteins without signal peptides is 2.0%. In the plants, there are 12 out of 91 (13%) against a background frequency of 0.64%, and in arthropods, there are 14 out of 44 (32%) against 2.98%. In fungi, there are only 4 ribosomal proteins out of 45 in the start codon intron set, but the overrepresentation is still significant (*p *< 10^-5^, χ^2^-test, *df *= 1). The descriptions of all start codon intron proteins from the GenBank data sets can be seen in Supplementary Table S2 [see Additional file 1]. In the genome sets, we found the same phenomenon: among human genes with start codon introns, there are 12.6% proteins marked "ribosomal" against a background frequency of 1.6%; in mouse, the frequencies are 15.1% against 1.3%, and in fly 15.5% against 1.7%. These differences are highly significant (*p *< 10^-15^, χ^2^-test, *df *= 1).

Since ribosomal proteins can be expected to be more conserved than other proteins, we investigated whether the higher conservation of proteins with start codon introns could be solely due to the high proportion of ribosomal proteins. The lists of RBHs were constrained to those where the human protein description did not contain the word "ribosomal," and the percent identities were compared again. The difference is still significant, although smaller (human vs. mouse with/without start codon introns: 89.6%/84.7%, two sample t-test, *p *< 1%; human vs. fly with/without start codon introns: 57.7%/39.7%, two sample t-test, *p *< 10^-14^).

## Discussion

When observing the start codon peak of introns in eukaryotic genes, the question naturally arose whether this was a biological phenomenon or some kind of artifact of gene finding. As mentioned in the description of the GenBank data sets, we discarded genes where the evidence was marked as "not experimental." However, we did not limit the data set to genes marked "/evidence=experimental," as this yielded too few genes to carry out the statistical analysis. The majority of genes do not have an "/evidence" qualifier at all. The GenBank data may therefore contain genes inferred by prediction.

In the whole-genome data, on the other hand, we discarded the genes solely based on prediction (those not included in RefSeq) and here, the start codon peak was still seen. This strongly suggests that the overabundance of start codon introns is a biological phenomenon. Also, the observation that proteins carrying a start codon intron comprise a special class points in this direction. We have shown that proteins with start codon introns are relatively short, lysine-rich, serine-poor and evolutionarily conserved. In addition, we have shown that the start codon intron positions are often conserved between human and fly. 

During the peer review process it was suggested that our method for showing the excess of phase 0 introns after the start codon could be questionable. The concern was that that the method of aligning the first 100 aa of all protein sequences, while showing the start codon peak, might fail to detect some other peaks. The reason is that insertions and deletions during evolution would alter the distance between start codon and intron peak, thereby spreading the peak over several positions. In other words, peaks in other positions have been "diluted" over evolution while the start codon peak is retained simply because we use the start codon as "anchor point" for our alignment. This "dilution effect" would be enhanced by the fact that the N-terminal parts of proteins are often less conserved than other parts, with many indels. The reviewer suggested that we specifically analyzed proteins that have their N-terminals conserved over evolution to avoid this suggested effect.

We did this by selecting from the list of RBHs between human and *Drosophila *those global alignments where neither the human nor the fly sequence had gaps within the first 20 positions. This yielded 661 pairs, of which 582 human sequences and 599 fly sequences were predicted to be without signal peptides. We then repeated the intron position statistics for these sequences, and the result is very clear: the phase 0 start codon peaks are even more conspicuous than those in Figure 3 (reaching frequencies of 0.062 for human and 0.053 for fly), and there are no peaks in other positions [see Supplementary Figure S3 from Additional file 1].

Furthermore, the fact that we can still see the phase 1 signal peptide peak in our start codon alignments despite the length variation of signal peptides suggests that the "dilution effect" does not completely wipe out peaks relatively close to the N-terminus, but only makes them broader. The reviewer suggested that the reason could be that proteins with signal peptides may have their N-terminals conserved over evolution, but this is not likely to be the case, since signal peptides are known to evolve rapidly [30].

The function of the start codon intron may be to allow the 5' UTR to participate in exon shuffling, so that different genes can exchange regulatory information. As described in the Background section, phase 0 introns are often found at the boundaries of evolutionary modules in proteins without signal peptides [22,26], and this effect is stronger in phylogenetically old proteins [23]. This would correspond quite well with the 5' UTR serving as an evolutionary module, separated from the protein coding domain by a phase 0 intron at the start codon, especially in well-conserved proteins.

If the start codon intron really is a boundary of an evolutionary module, one should expect that the second intron would often be a phase 0 intron, too, since modules in exon shuffling most often have introns of the same phase in both ends [26]. We tried to investigate this by counting the phases of the second introns in proteins where the first intron was a start codon intron, compared with proteins where the first intron was a phase 0 intron in another position. In vertebrates and plants, there was indeed a tendency towards a higher proportion of second phase 0 introns for the start codon intron set, but it was not significant. In arthropods there was no difference, and in fungi it was in fact lower (though not significantly).

Another explanation for the start codon introns could be that the nucleotide context of codons 1 and 2 provide a proto-splice site. The codon before is necessarily "atg," so the two nucleotides before the intron is "tg" instead of the canonical "ag." However, the "a" is not very well conserved, and "t" is the next most common nucleotide in the -2 position relative to the intron [12,18]. Also, in the evolutionarily conserved amino acids examined by Sverdlov *et al*. [16] in order to reconstruct ancestral proto-splice sites (see description in the Background section), Methionine was slightly overrepresented immediately before a phase 0 intron. Consequently, the start codon could be part of a proto-splice site if the first position of codon 2 is a "g".

Indeed, "g" is enriched in position +4 (relative to the translation start site) in eukaryotes [31]. However, this enrichment is much higher in plants than in vertebrates or *Drosophila *[32], so if this were to be the sole explanation for start codon introns, we should expect a much stronger start codon intron signal in plants than in animals, and this is not the case (on the contrary, the signal is weaker in plants, see Figure 2). On the other hand, the relatively weak start codon intron signal in plants could be explained within the proto-splice site model if there is a much higher frequency of proto-splice sites in the plant genes than in the vertebrate or arthropod genes as a whole. We have not checked whether this is the case.

The 5' bias we observed in the position of introns within the non-vertebrate genes is in contrast to Mourier and Jeffares [3], who reported that introns in multicellular genomes are evenly distributed throughout genes. Our fungal data set does contain unicellular organisms, but arthropods and plants do not. On the other hand, Sakurai *et al*. and Lin and Zhang [2,4] did find a 5' bias in virtually all genomes studied. Interestingly, they even found 5' bias in human and mouse, where we did not observe it.

The fact that ribosomal proteins are strongly overrepresented in start codon intron proteins is striking, because it is an example of an intron position being conserved in a *functional *class of proteins that are not related by descent (the data sets were strictly homology reduced). To be absolutely sure our homology reduction procedure had not let any pairs of homologous sequences through, we performed an all-*versus*-all global alignment of the sequences with start codon introns in all the four GenBank organism groups. No pairs showed more than 28.5% identity in the global alignment. We also made phylogenetic trees of the start codon intron sets and found no clusters (the trees look just like stars). Plots of % identity *versus *alignment score and the trees can be seen in Supplementary Figure S4, section 1 [see Additional file 1]. 

Another example of this is the position 5 phase 0 peak in *Drosophila*. Initially, we thought that it could be a gene finding artifact, since it was specifically seen in one genome. However, the extremely high proportion of cuticle constituents convinced us that this too is a biological phenomenon. It is also not entirely unknown in the literature: Rondot *et al*. [33], while describing two larval-pupal cuticle protein genes from the insect *Tenebrio molitor*, found that both had a single intron within the signal peptide coding region (they do not write the exact location and phase of those two introns, but from a figure in the paper it can be inferred that they are indeed phase 0 introns, at positions 4 and 5). In addition to their own observation, they remarked that such an intron localization (*i.e*. a single intron within the signal peptide region) has been described in cuticle protein genes from many different species, including *Drosophila*. Again, note that this is not simply a gene family with a conserved intron position, since the peak is also seen in the heavily homology-reduced set. In order to test this further, we did an all-*versus*-all global alignment of the 20 sequences in the arthropod peak and found no pairs with more than 32.1% identity [see Supplementary Figure S4, section 2, from Additional file 1].

## Conclusion

The start codon peak of phase 0 introns is so conspicuous that it is quite remarkable that it has only been described in a single sentence in the literature before. As mentioned above, Yoshihama *et al*. [29] saw the phenomenon in human ribosomal proteins, but to the best of our knowledge we are the first to provide a general investigation of it. In this paper, we have filled this gap in the description of intron positions in genes and argued that this phenomenon seems to be a biological reality and not simply an artifact of gene finding or alignment.

## Methods

### GenBank data

Data sets were extracted from GenBank release 149.0 [34]. Vertebrate data were extracted from the GenBank divisions gbpri, gbrod, gbmam and gbvrt. Arthropod data were taken from gbinv, while Fungi and flowering plants (*Magnoliophyta*) were both found in gbpln. Observe that *Magnoliophyta *comprises both monocots and dicots.

Information about introns in the sequences were taken from "CDS" feature lines with a "join(...)" function in the feature position. "CDS" features on the opposite strand, marked by "complement(join(...))", were also parsed.

Before analysis, the GenBank entries went through a filtering process. Genes were skipped for the following reasons (listed in the order the tests were applied): if the gene was a pseudogene (indicated by the qualifier "/pseudo"); if the gene was marked "/evidence=not_experimental"; if the sequence was incomplete, indicated by ">" or "<" in the positions; if the argument to "join" contained a cross-reference to another entry (in this case, it was not possible to assess the length of the intron); if the argument to "join" contained "complement" (this was a rare occurrence that we did not bother to parse); if the amino acid sequence contained "X" (unknown amino acid) or "U" (selenocysteine); if the amino acid sequence lacked an initial methionine; if the gene contained introns or exons of zero or negative length; if there was an annotated "gap" feature within the region of the "CDS" feature; or if the length of the amino acid sequence did not match the length inferred from the argument to "join".

At this point, the data were used for calculating intron length statistics. After getting the results from that analysis, two more filtering steps were applied: genes with an intron shorter than the found cutoff were skipped, as were genes with introns that did not conform to the splice site consensus "gt...ag".

Non-consensus splice sites are, as described in the Background section, known to exist, but they are rare. Burset *et al*. [10] estimated that at least half the annotated non-canonical splice sites should be annotation errors. It would be interesting to know whether "at...ac" introns follow the same positional distribution, but they are simply too rare to make this kind of statistics.

The number of genes discarded for the various reasons can be seen in Table 2. The script for GenBank parsing was written in PERL.

**Table 2:** GenBank data sets

Organism group	Vertebrata	Arthropoda	Fungi	Magnoliophyta
Total CDS with introns	54729	34336	31441	95711

pseudogene	1899	90	101	789
not experimental	1204	515	504	9150
incomplete 5' end (<)	15622	10583	11143	11659
incomplete 3' end (>)	5417	1664	569	1561
cross-reference	10445	231	2	60
join (complement)	0	16	0	34
contains 'X'	106	120	71	100
contains 'U'	26	4	0	0
no initial 'M'	222	51	9	34
zero or negative length	36	7	17	35
annotated gap	480	6	0	25
length mismatch	466	19	11	18

Used for length statistics	18807	21030	19014	72247

non-gt...ag	1734	818	1159	3368
intron too short	550	1244	2354	12125

CDS accepted	16523	18968	15501	56754

After homology reduction	3542	4179	4525	12751

With signal peptides	755	769	431	1051
Without signal peptides	2552	3202	3814	10370

The number of genes (CDS features) found in GenBank within the four organism groups studied. The number of genes discarded for various reasons. The number kept after homology reduction. The numbers predicted to contain or not to contain a signal peptide.

Functional annotation of the proteins in the GenBank data sets were extracted from GenBank by searching for the "/product" qualifier. In general, the vertebrate set is much more annotated than the other organism groups, having a protein name in 74% of the "CDS" features. The corresponding figures for fungi and plants are 40% and 19%. For arthropods, the situation is more complicated, since functional annotation for *Drosophila *entries is often not found in GenBank itself – the "/product" qualifier contains a code such as "CG4111-PA" – but can be found via cross-referencing to the NCBI Gene database, see below. A protein sequence without a functional annotation can be marked in several different ways: by having no "/product" qualifier, by having the value "unknown," or by having the value "hypothetical protein" or "putative protein."

The GenBank data sets are provided for download at our website [35].

### Whole-genome data

Genomic data for human (*Homo sapiens*), mouse (*Mus musculus*) and fly (*Drosophila melanogaster*), was downloaded from NCBI (National Center for Biotechnology Information) [36] in the following versions: Human: Build 35; Mouse: Build 35; Fly: April 2005 build (contains the annotation from FlyBase Release 4.1 and 3.2).

In all cases, the chromosomal GenBank format files were parsed using the FeatureExtract software [37] to collect both DNA sequence and Intron/Exon annotation for all "CDS" regions.

For the human and mouse datasets, the following cleaning strategy was followed: Only genes whose protein-product is represented in the curated RefSeq database [38] (name starts with "NP_") were kept, and all genes which contained the phrases "translation discrepancy" and "hypothetical protein" in the "CDS" feature note were discarded. For the *Drosophila *genome, all genes with ambiguous exon positions were removed, as were genes with "codon_start" annotated to be 2 or 3.

Following the initial cleaning all genes were translated using the Virtual Ribosome software [39] using the software's option of creating an annotation string describing the phase and position of the underlying introns. Protein sequences from genes with multiple in-frame stop-codons were discarded. For all three genomes, alternative splicing was handled simply by renaming each alternative spliced transcript as "XXX_splic1", "XXX_splic2", etc.

After cleaning, the human genome set contained 13916 sequences, the mouse set 13957 sequences, and the fly set 19297 sequences.

Functional annotation (GO categories and descriptive annotation) was derived directly from the "CDS" comments (with the "/product" qualifier) in the case of human and mouse, and by cross-referencing the GeneID to the NCBI Gene database [40] in the case of *Drosophila*.

For the results presented in Supplementary Figure S2 [see Additional file 1], we removed ribosomal proteins by skipping all entries where the comment (mouse and human) or GO annotation (fly) contained the string "ribosom" This removed 247 sequences from the human set, 209 from the mouse set, and 290 from the fly set.

The whole-genome data sets are provided for download at our website [35].

### Homology reduction

Homology reduction of the GenBank data sets was carried out in order to avoid gene families with conserved intron positions showing up as peaks in the intron position plots.

All the amino acid sequences in each data set were aligned to each other using BLAST version 2.2.9 [41]. Filtering of low-complexity sequences was turned off.

**Figure 5:**
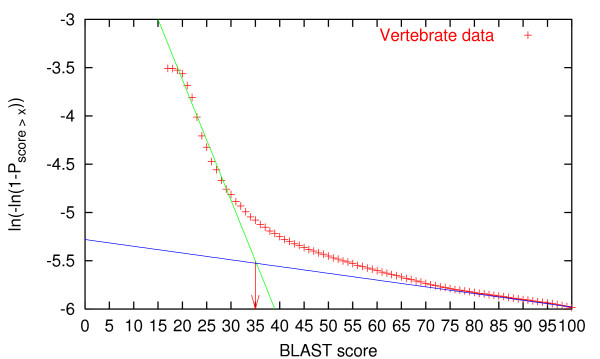
Finding the threshold for homology reduction: the cumulated score distribution is plotted in an extreme value plot, and the threshold is set to the score value where the two lines intersect. Here shown for the vertebrate data set.

The threshold was found in the same way as for the translation initiation site dataset used in developing NetStart [42]. Briefly, the BLAST score was plotted against an inverse cumulated extreme value distribution: *f*(*x*) = ln(-ln(1 - *P*_*score*≥*x*_)). Alignment scores for unrelated sequences are expected to follow an extreme value distribution [43] and so show up as a straight line in such a plot. The idea is then to put the threshold where the plot deviates from a straight line. In this case, the curve did not have a well-defined "kink" but rather smoothly arched away from the straight line, so the cutoff was placed approximately where a straight line drawn through the higher scores crossed the straight line for lower scores, see Figure 5. This yielded a threshold of 35 bits (BLAST score) for all four data sets.

Note in Figure 5 that there is also a deviation from the straight line for very low scores (<20). This is due to the fact that the alignments were made using BLAST instead of a full alignment of all sequences to each other. There are fewer very low scores than expected because they simply do not show up in the BLAST output.

After finding the threshold, the removal of homologous sequences was carried out using algorithm 2 of Hobohm *et al*. [44]. The cutoff of 35 in BLAST score corresponded to 0.003–0.8 in E-value. This turned out to be a very stringent threshold, weeding out 71–79% of the sequences. The numbers of genes retained after homology reduction are given in Table 2.

To further ensure that no protein families have escaped our homology reduction, we analyzed the sequences in the start codon peaks and the arthropod position 5 peak in the following way: For each dataset, all protein sequences were pairwise globally aligned against the rest of the set using the "align" program from the FASTA package [45], and alignment score and percent idendity were plotted. Furthermore, we used ClustalW [46] to construct a "phylogenetic" tree based on pairwise distances (ClustalW's guide-tree), and visualized the relationship by plotting the tree with "unrooted" [47]. These results are shown in Supplementary Figure S4 [see Additional file 1].

The whole-genome data were not homology reduced.

### Signal peptide prediction

Signal peptide prediction was done with SignalP v. 3.0 [48]. Sequences were placed in the categories according to the D-score of SignalP-NN and the prediction of SignalP-HMM. In cases where SignalP-NN and SignalP-HMM did not agree, the sequence was disregarded. The numbers of proteins predicted to be with and without signal peptides can be seen in Table 2.

### Reciprocal Best Hits

Conservation of proteins were investigated using the whole-genome sets. All human sequences were aligned to all mouse and fruit fly sequences using BLAST [41]. For each human gene, the best hit in the database of mouse or fly was saved, and *vice versa*. If the best hit for gene A in human was gene B in mouse or fly, and the best hit for gene B simultaneously was gene A, a Reciprocal Best Hit (RBH) between gene A and gene B was recorded. 8089 RBHs were found between human and mouse, and 4223 between human and fly. The pairs of proteins involved in each RBH were then globally aligned using the program "align" from the FASTA package [45] in order to calculate the overall percent identity and investigate whether intron positions were conserved.

### Statistical tests

Statistical tests were done in R [49]. When doing χ^2^-tests, Yates' continuity correction was used for 2-by-2 contingency tables. Two-sample t-tests were always preceded by an F-test to compare the two variances; if the variances were significantly unequal, Welch test was used.

## Authors' contributions

The project idea was conceived by HN, who also made all initial work on the GenBank data, the statistical analysis, and the main discoveries. RW provided and cleaned the genomic data, did the all-vs-all alignment analysis and set up the supplementary material. The manuscript was drafted by HN with RW providing details about the genomic analysis. The manuscript was continuously discussed and evaluated by both authors.

## Supplementary Material

Additional file 1 Additional file 1 which has been uploaded with this manuscript is a PDF document of 3.3 MB (17 pages). It contains Supplementary Figures S1–S4 and Supplementary Tables S1 and S2 which have been referred to in the text. The data sets used in this study are deposited at our website [35], where you can also find a web page version of the supplementary figures and tables. Click here for file
