# Supplementary material for: An overabundance of phase 0 introns immediately after the start codon in eukaryotic genes
Source: BMC Genomics. 2006 Oct 11;7:256. doi: 10.1186/1471-2164-7-256 (PMC1626468; doi:10.1186/1471-2164-7-256)
Supplement: Additional file 1 — which has been uploaded with this manuscript is a PDF document of 3.3 MB (17 pages). It contains Supplementary Figures S1–S4 and Supplementary Tables S1 and S2 which have been referred to in the text. The data sets used in this study are deposited at our website [35], where you can also find a web page version of the supplementary figures and tables. [file 1471-2164-7-256-S1.pdf]

An overabundance of phase 0 introns immediately after the start codon in eukaryotic genes - *supplementary material*

Henrik Nielsen* and Rasmus Wernersson

Center for Biological Sequence Analysis, Technical University of Denmark, Building 208, 2800 Lyngby, Denmark

Email: Henrik Nielsen*- hnielsen@cbs.dtu.dk; Rasmus Wernersson - raz@cbs.dtu.dk;

*Corresponding author

Data sets

All data sets are available for download at: http://www.cbs.dtu.dk/suppl/introns/

References

1. Pearson WR, Lipman DJ: **Improved tools for biological sequence comparison.** *Proc Natl Acad Sci U S A* 1988, **85**(8):2444–2448.
2. Thompson JD, Gibson TJ, Plewniak F, Jeanmougin F, Higgins DG: **The CLUSTAL_X windows interface: flexible strategies for multiple sequence alignment aided by quality analysis tools.** *Nucleic Acids Research* 1997, **25**(24):4876–4882.
3. **Unrooted**. *[http://pbil.univ-lyon1.fr/software/unrooted.html]*.

Figure S1: intron length distribution for introns up to 250 nt

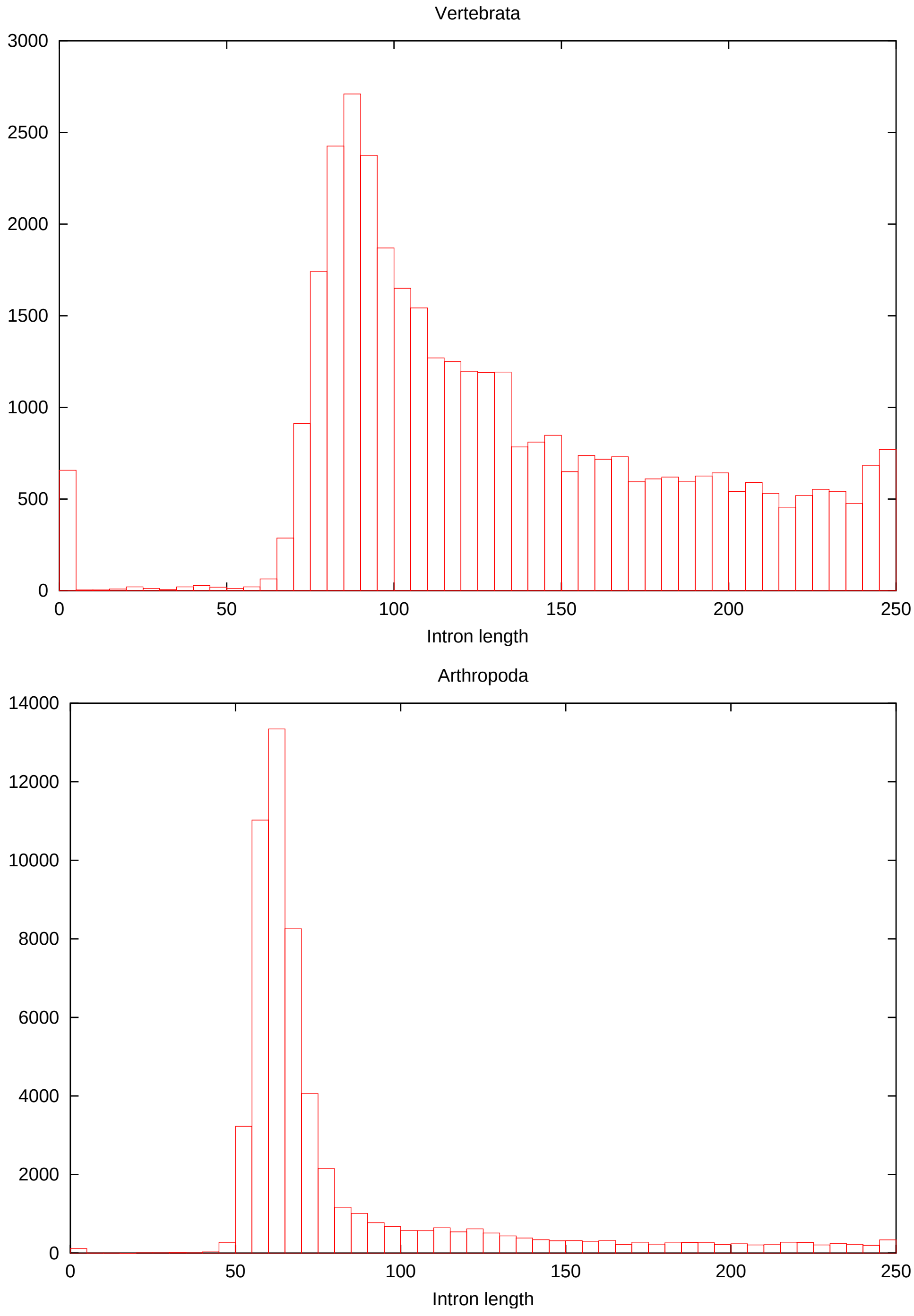

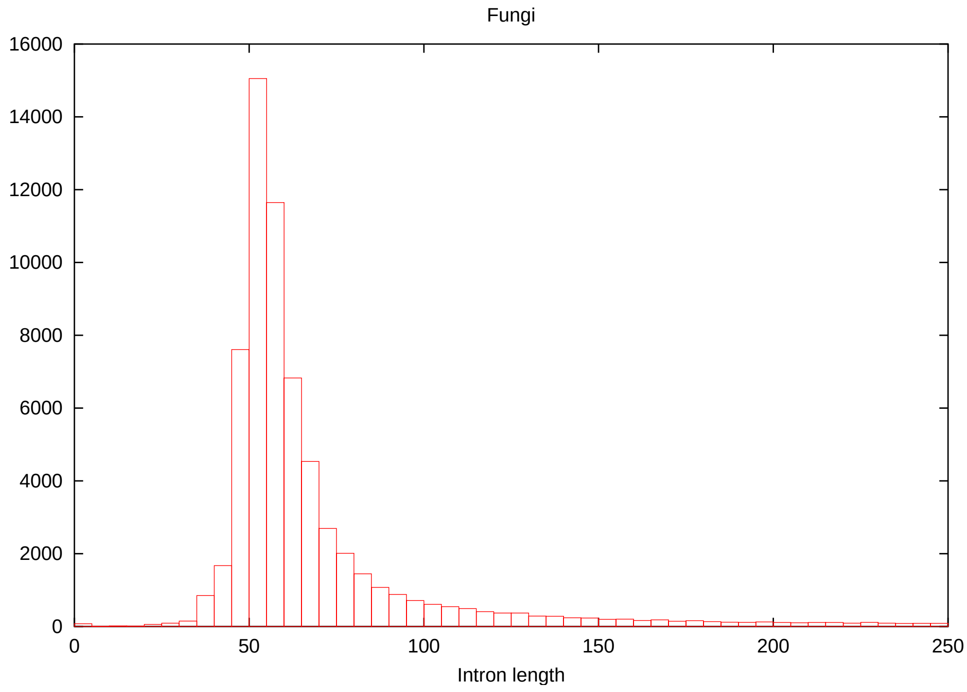
Fungi
16000
14000
12000
10000
8000
6000
4000
2000
0
0
50
100
150
200
250
Intron length

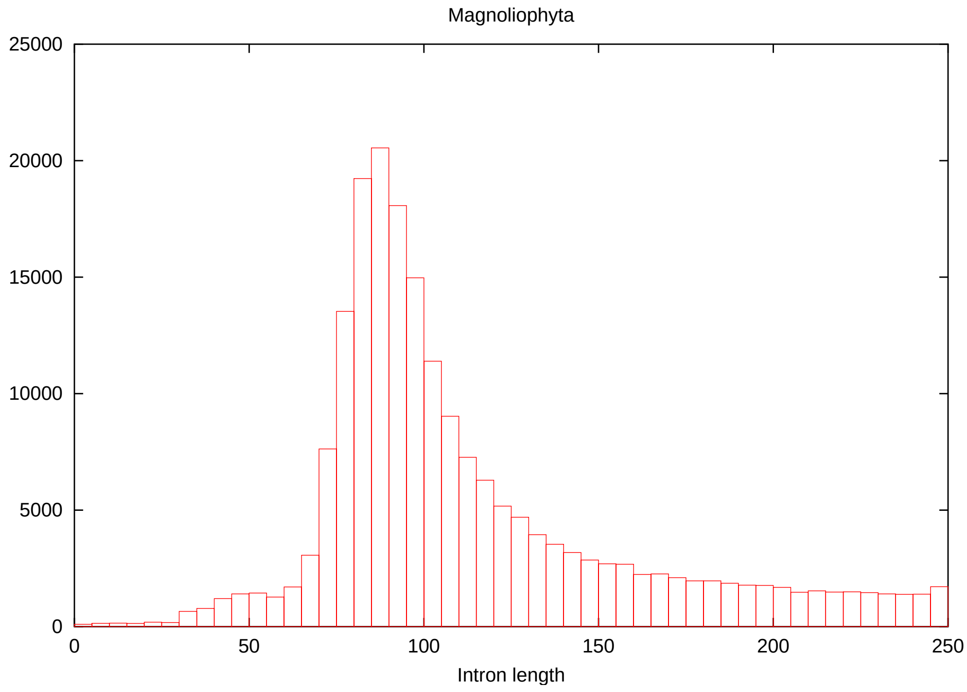
Magnoliophyta
25000
20000
15000
10000
5000
0
0
50
100
150
200
250
Intron length

Figure S2: Intron position statistics for genome data without ribosomal proteins

In order to show that the phenomenon of start codon introns is not limited to ribosomal proteins, we have calculated the distribution of intron positions for the whole-genome data sets (for proteins without signal peptides) with ribosomal proteins removed. This figure should be compared to the right half of Figure 3 in the paper.

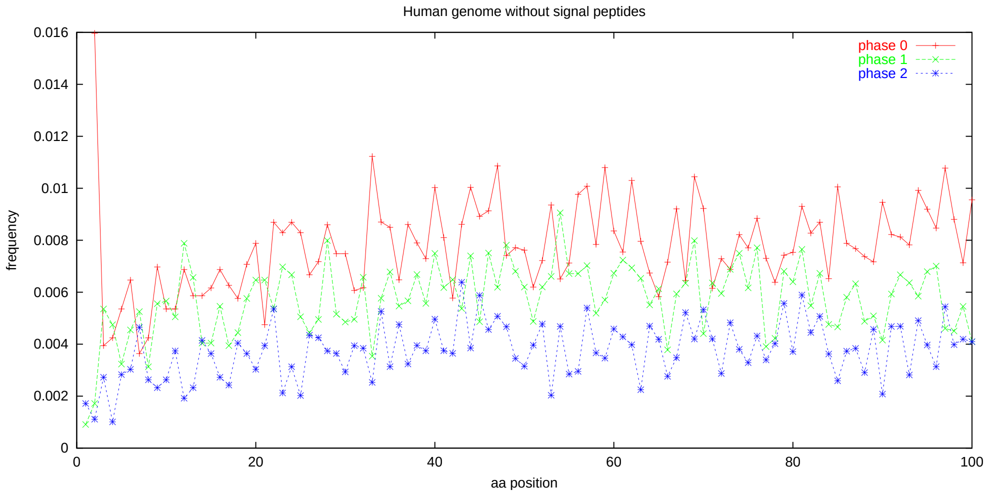
Human genome without signal peptides
frequency
aa position
phase 0
phase 1
phase 2
0
0.002
0.004
0.006
0.008
0.01
0.012
0.014
0.016
0
20
40
60
80
100

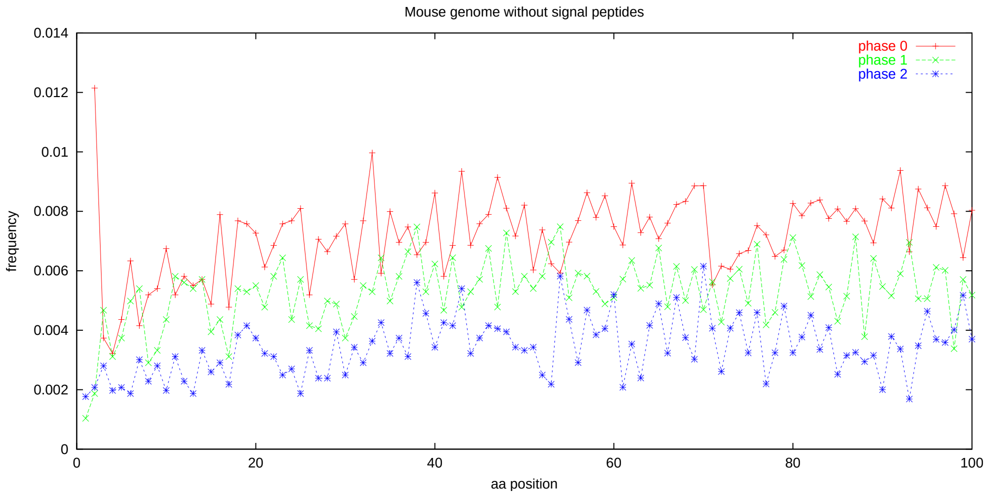
Mouse genome without signal peptides
frequency
aa position
phase 0
phase 1
phase 2
0
0.002
0.004
0.006
0.008
0.01
0.012
0.014
0
20
40
60
80
100

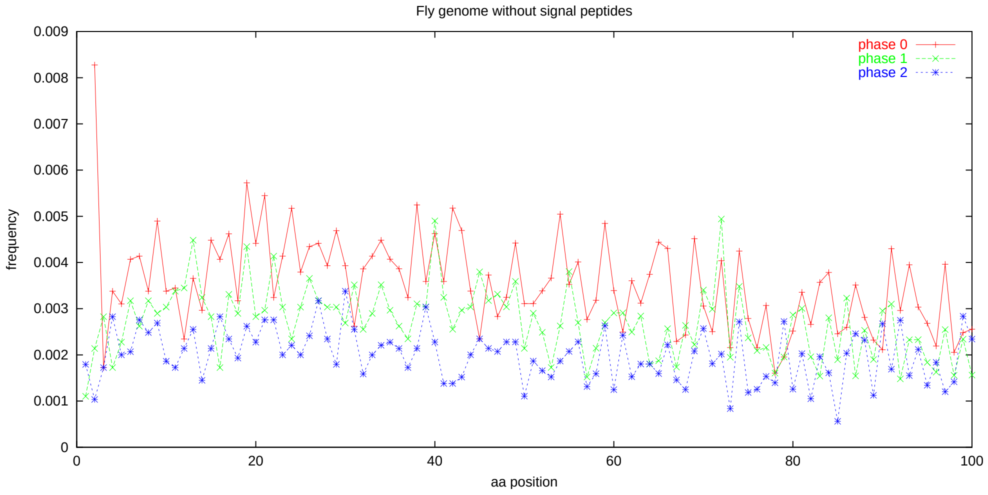
Fly genome without signal peptides
frequency
aa position
phase 0
phase 1
phase 2
0
0.001
0.002
0.003
0.004
0.005
0.006
0.007
0.008
0.009
0
20
40
60
80
100

Figure S3: Intron position statistics for human and Drosophila proteins with conserved N-terminals

In order to show that the start codon peak is also present in proteins with few indels in the N-terminal part, we selected from the list of RBHs (Reciprocal Best Hits) between human and Drosophila those global alignments where neither the human nor the fly sequence had gaps within the first 20 positions. This yielded 661 pairs, of which 582 human sequences and 599 fly sequences were predicted to be without signal peptides.

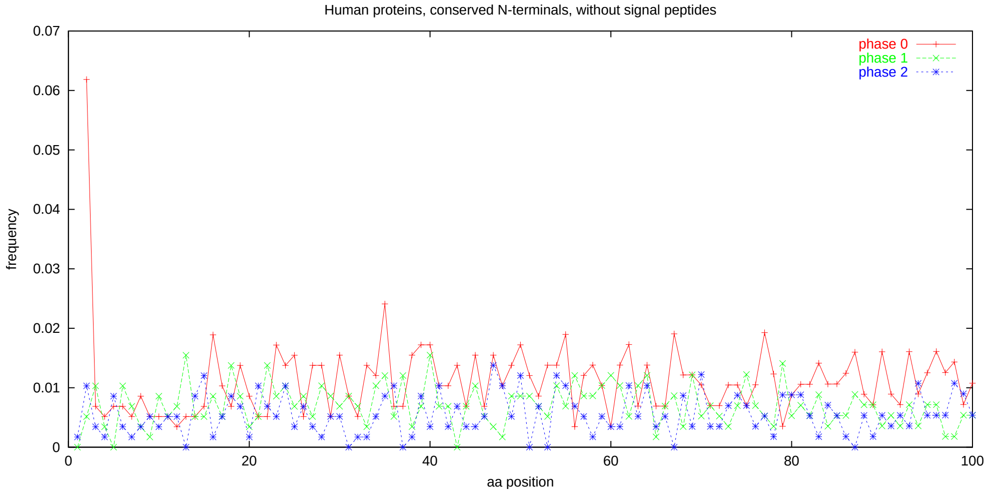

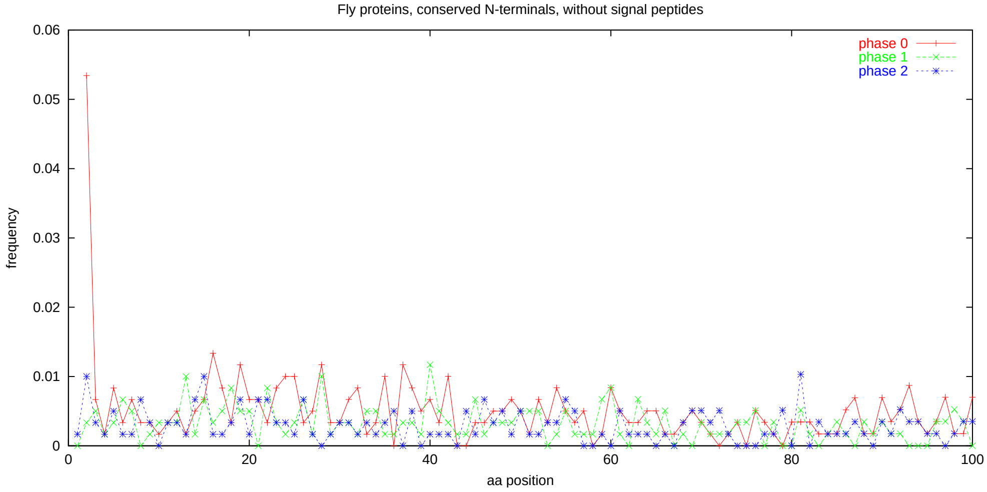

Figure S4: All vs. all alignments: Score/Identity Plots and distance trees

In order to make absolutely sure that the start codon and position 5 peaks, were not an artefact due to homologous proteins (even though the datasets have been homology reduced), we have analyzed the data the following way:

For each dataset, all protein sequences where aligned pairwise against the rest of the set using the ALIGN program [1], and alignment score and percent idendity were plotted. Furthermore, we used CLUSTALW [2] to construct a "phylogenetic" tree based on pairwise distances (CLUSTALW's guide-tree), and visualized the relationship by plotting the tree with "`UNROOTED`" [3].

As seen in Section 1 & 2 below, the proteins in both the start codon peak and position 5 peak, are clearly not related. For reference we did the same analysis on 100 randomly selected proteins from the non-homology reduced Vertebrate set, and as seen in Section 3, it is clearly seen that a number of these sequences are related by homology.

Section 1: Start codon peaks

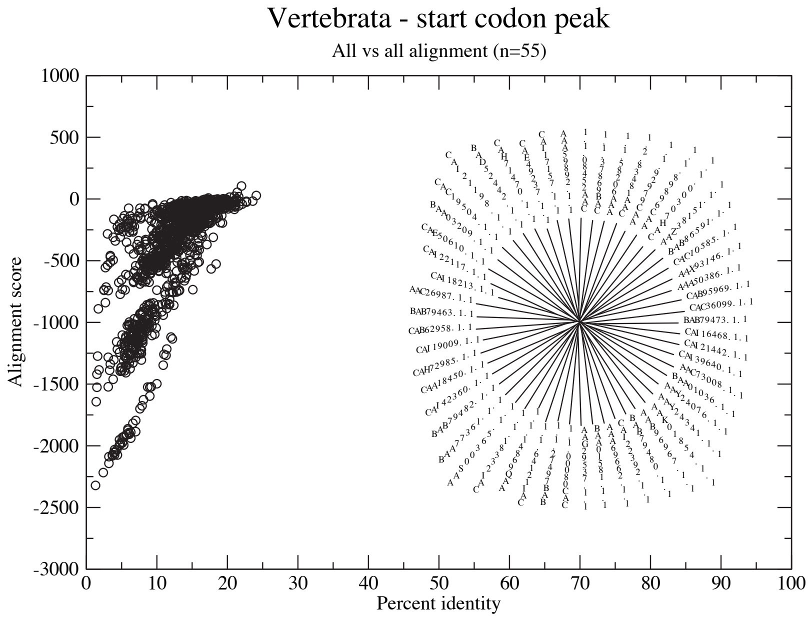

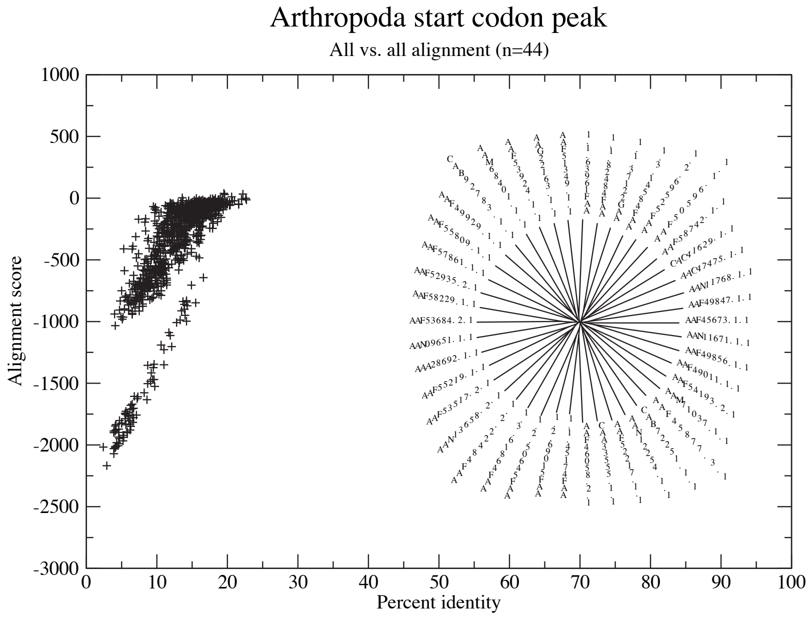

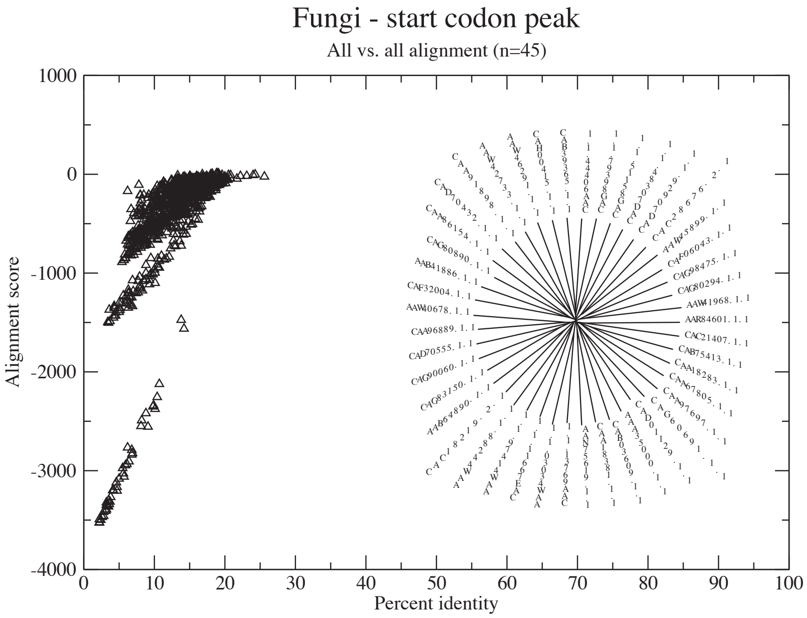
Fungi - start codon peak
All vs. all alignment (n=45)
Alignment score
Percent identity
1000
0
-1000
-2000
-3000
-4000
0 10 20 30 40 50 60 70 80 90 100

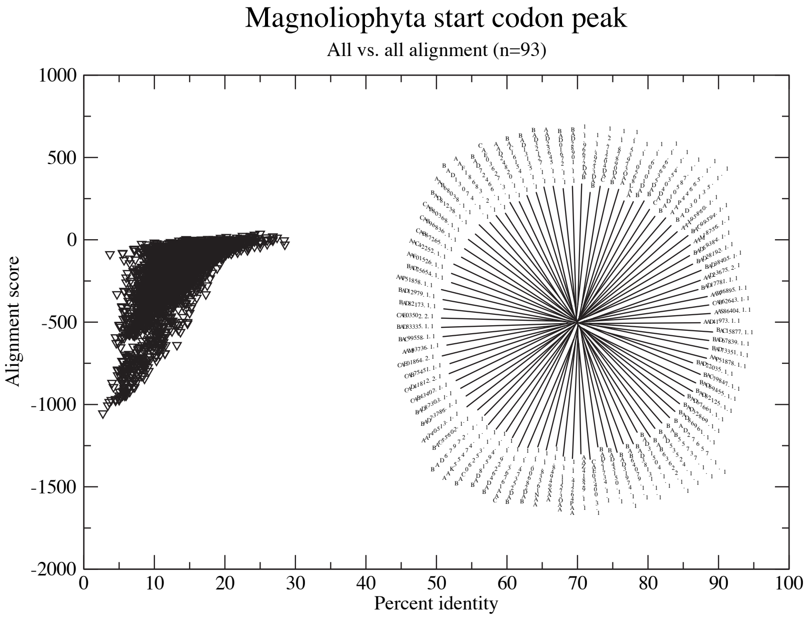
Magnoliophyta start codon peak
All vs. all alignment (n=93)
Alignment score
Percent identity
1000
500
0
-500
-1000
-1500
-2000
0 10 20 30 40 50 60 70 80 90 100

Section 2: Arthropoda position 5 peak

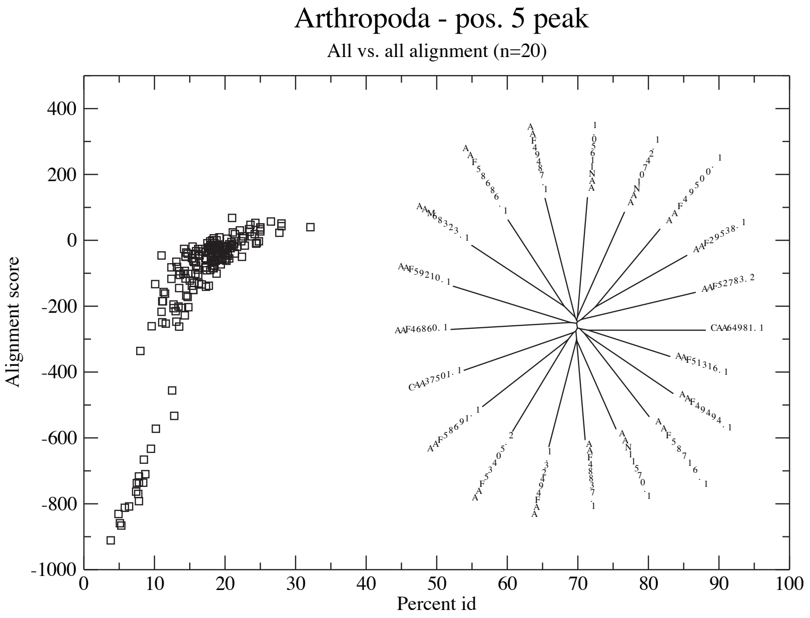

Section 3: Reference plot of non-homology reduced data

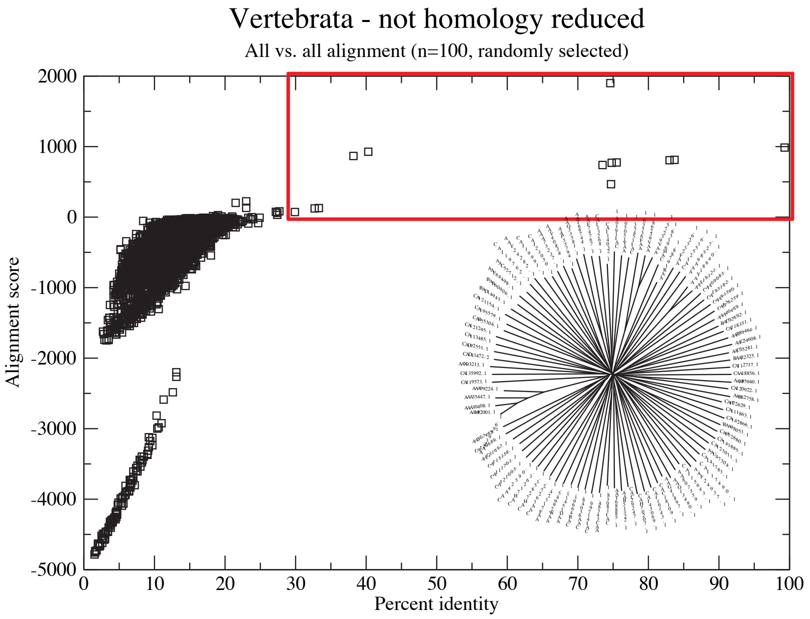

Table S1: lengths, nucleotide frequencies and dinucleotide frequencies for start codon introns compared to other first phase 0 introns

Length and nucleotide statistics for start codon introns ("**sci**") and other phase 0 first introns ("**other**"). Where the difference for a particular dinucleotide is greater than 0.5%, the higher percentage is shown in boldface.

When examining the nucleotide distrution, significant differences are found for vertebrates, fungi, and plants ($p < 10^{-4}$, χ^2-test, $df = 3$), but not for arthropods. However, when using nucleotide pair frequences, significant differences are found for all four groups ($p < 10^{-3}$, χ^2-test, $df = 15$). The nucleotide pair frequences are shown in Table S1 (above). However, there does not seem to be any dinucleotide preferences that are the same in all four organism groups.

	Vertebrata		Arhropoda		Fungi		Magnoliophyta	
	sci	no sci	sci	no sci	sci	no sci	sci	no sci
Length statistics								
Mean	3059.6	4941.3	631.0	822.4	170.0	106.6	498.6	478.8
P-val	0.01681		0.4927		0.008802		0.738	
Nucleotide statistics								
**a**	26.20%	26.95%	29.27%	29.28%	29.16%	26.72%	28.17%	27.98%
**c**	21.05%	20.75%	19.90%	20.27%	19.93%	21.63%	19.94%	19.36%
**g**	23.48%	22.11%	19.84%	19.83%	20.61%	21.30%	19.42%	20.29%
**t**	29.18%	30.18%	30.99%	30.63%	30.31%	30.35%	32.46%	32.37%
Dinucleotide statistics								
**aa**	7.85%	8.27%	11.01%	10.52%	**9.05%**	7.65%	9.20%	8.91%
**ac**	4.59%	4.66%	4.74%	5.09%	5.58%	5.87%	4.84%	4.77%
**ag**	7.25%	7.09%	4.91%	5.08%	5.95%	5.82%	5.36%	5.48%
**at**	6.50%	6.93%	8.66%	8.62%	**8.76%**	7.64%	8.83%	8.88%
**ca**	6.59%	6.67%	6.50%	6.73%	6.44%	6.45%	5.98%	5.93%
**cc**	5.57%	5.52%	4.65%	4.50%	4.04%	**4.89%**	4.38%	4.25%
**cg**	1.69%	1.32%	3.64%	3.69%	3.26%	3.69%	3.14%	3.08%
**ct**	7.20%	7.25%	5.14%	5.36%	6.30%	6.80%	6.48%	6.14%
**ga**	5.95%	5.85%	4.99%	5.09%	6.34%	5.90%	5.43%	5.63%
**gc**	5.13%	4.68%	5.29%	5.19%	4.30%	4.39%	4.11%	4.23%
**gg**	**6.64%**	5.87%	3.91%	4.11%	3.99%	3.88%	4.11%	4.39%
**gt**	5.72%	5.69%	5.52%	5.34%	5.51%	**6.38%**	5.61%	5.86%
**ta**	5.79%	6.16%	6.82%	6.97%	**7.50%**	6.98%	7.62%	7.56%
**tc**	5.75%	5.90%	5.25%	5.51%	6.13%	**6.68%**	6.64%	6.15%
**tg**	7.87%	7.82%	7.25%	6.84%	6.94%	7.17%	6.66%	**7.17%**
**tt**	9.76%	**10.31%**	11.71%	11.34%	9.92%	9.82%	11.60%	11.55%

Table S2: Protein names or GO categories (for arthropoda) for all the start codon intron proteins

Vertebrata - GenBank dataset, homology reduced

Locus	Protein Id	"Product" notes
AB020236	BAA77361.1	*"ribosomal protein L27A"*
AB040440	BAB86591.1	*"glia maturation factor gamma"*
AB061825	BAB79463.1	*"ribosomal protein L18"*
AB061834	BAB79472.1	*"ribosomal protein L37"*
AB061835	BAB79473.1	*"ribosomal protein L39"*
AB061842	BAB79480.1	*"ribosomal protein S20"*
AB061844	BAB79482.1	*"ribosomal protein S25"*
AB191468	BAD52442.1	*"hypothetical protein"*
AC004854	AAS00365.1	*"unknown"*
AC016753	AAY24341.1	*"unknown"*
AC020550	AAX93146.1	*"unknown"*
AC073341	AAQ96841.1	*"unknown"*
AC132479	AAY24076.1	*"unknown"*
AF041427	AAB96967.1	*"ribosomal protein s4 Y isoform"*
AF082027	AAC97929.1	*"alpha tubulin"*
AF100956	AAC69898.1	*"ribosomal protein subunit S18"*
AF229836	AAK01854.1	*"vesicle-associated membrane protein 5"*
AF305057	AAG29537.1	*"RTS beta"*
AL136181	CAH72985.1	*"transmembrane protein 10"*
AL137067	CAC08000.1	*"Sec61 beta subunit"*
AL139289	CAI23381.1	
AL157783	CAI12146.1	*"cAMP responsive element modulator"*
AL354928	CAI39640.1	*"ribosomal protein L35"*
AL355815	CAC19504.1	
AL357314	CAI22392.1	*"Rab geranylgeranyltransferase, beta subunit"*
AL359454	CAI17157.1	*"chromosome 13 open reading frame 12"*
AL365338	CAH70300.1	*"spermatid perinuclear RNA binding protein"*
AL451000	CAH71470.1	*"novel protein (MDS2)"*
AL512628	CAI16468.1	*"ribosomal protein S24"*
AL671762	CAI18213.1	*"LSM2 homolog, U6 small nuclear RNA associate"*
AL844518	CAE50610.1	*"novel protein similar to human and mous"*
AL928906	CAE49237.1	*"novel protein simlar to human and mouse vacuola"*
AL929220	CAI21198.1	*"enhancer of rudimentary homolog (Drosophila)"*
CHKRIG	BAA01036.1	*"ribosomal protein S15"*
CHKRPL37A	BAA03209.1	*"ribosomal protein L37a"*
CHKRPL5G	BAA01581.1	*"ribosomal protein L5"*
CIUEF2G	AAA50386.1	*"elongation factor 2"*
DQ118138	AAZ38151.1	*"interferon-stimulated protein 17/ubiquiti"*
HS1054A22	CAI19009.1	
HS164F3	CAI42360.1	*"ribosomal protein L36a"*
HS333H23	CAA18450.1	
HS395P12	CAA18438.2	*"tumor necrosis factor (ligand) superfamily"*
HS747E2	CAB62958.1	
HSA289880	CAB96873.1	*"leucine zipper transcription factor-like 1"*
HSA462D18	CAC10585.1	*"destrin (actin depolymerizing factor)"*
HSDJ636H5	CAI22117.1	*"RAD54-like (S. cerevisiae)"*
HSJ468K18	CAI21442.1	*"chromosome 6 open reading frame 93"*
HSU29895	AAC73008.1	*"4-hydroxyphenylpyruvate-dioxygenase"*
HSU41448	AAC26987.1	*"ribosomal protein S26"*
HUMMYLCA	AAA59892.1	*"smooth muscle myosin light chain"*
HUMPCBD	AAA69662.1	*"pterin-4a-carbinolamine dehydratase"*
HUMRPS17A	AAA60285.1	
MMU272272	CAB95969.1	*"20S proteasome subunit C2"*
RNMYOLC1	CAA25480.1	*"MLC2"*
TRU310912	CAC36099.1	*"putative ribosomal protein L14"*

Arthropoda - GenBank dataset, homology reduced

Locus	Protein Id	"Product" notes	GO Term
AE003422	AAF45673.1	*"CG14813-PA"*	"[ISS]:retrograde transport, Golgi to ER"
AE003427	AAF45877.3	*"CG14271-PB"*	"[ISS]:sperm motility"
AE003435	AAF46058.2	*"CG4111-PA, isoform A"*	"[ISS]:cytosolic large ribosomal subunit"
AE003456	AAF46816.3	*"CG5625-PA, isoform A"*	"[ISS]:vesicle-mediated transport"
AE003462	AAF47154.1	*"CG3997-PA"*	"[ISS]:cytosolic large ribosomal subunit"
AE003491	AAN09651.1	*"CG1903-PA, isoform A"*	"[IEA]:helicase activity"
AE003498	AAF48422.2	*"CG15027-PA"*	
AE003498	AAF48428.1	*"CG9091-PA"*	"[ISS]:cytosolic large ribosomal subunit"
AE003500	AAF48541.3	*"CG33180-PB"*	"[IEA]:protein transporter activity"
AE003513	AAF49011.1	*"CG14233-PA"*	"[IEP]:mesoderm development"
AE003519	AAN11671.1	*"CG32027-PA"*	"[ND]:biological process unknown"
AE003529	AAN11768.1	*"CG6151-PC, isoform C"*	
AE003539	AAF49847.1	*"CG11276-PA, isoform A"*	"[ISS]:unfolded protein binding"
AE003539	AAF49856.1	*"CG11267-PA"*	"[ISS]:cytosolic small ribosomal subunit"
AE003541	AAF49929.1	*"CG10418-PA"*	"[ISS]:nuclear mRNA splicing, via spliceosome"
AE003542	AAN12254.1	*"CG17146-PB, isoform B"*	"[ISS]:uridine kinase activity"
AE003559	AAF50596.1	*"CG8615-PA"*	"[ISS]:cytosolic large ribosomal subunit"
AE003579	AAF51096.2	*"CG33123-PA"*	"[ISS]:leucine-tRNA ligase activity"
AE003585	AAF51349.1	*"CG17158-PA"*	"[ISS]:F-actin capping protein complex"
AE003608	AAF52217.1	*"CG5827-PA, isoform A"*	"[ISS]:cytosolic large ribosomal subunit"
AE003619	AAF52596.2	*"CG7424-PA"*	"[ISS]:cytosolic large ribosomal subunit"
AE003628	AAF52935.2	*"CG5362-PA"*	"[ISS]:L-malate dehydrogenase activity"
AE003650	AAF53517.2	*"CG5869-PA"*	"[ISS]:actin binding"
AE003659	AAF53684.2	*"CG15162-PA"*	
AE003668	AAF53924.1	*"CG9324-PA"*	"[NAS]:proteasome complex"
AE003677	AAF54193.2	*"CG9667-PA"*	
AE003690	AAF54605.2	*"CG6684-PB, isoform B"*	"[ISS]:cytosolic small ribosomal subunit"
AE003708	AAN13658.2	*"CG5044-PB, isoform B"*	"[ISS]:3-hydroxyisobutyryl-CoA hydrolase activity"
AE003710	AAF55219.1	*"CG4525-PA"*	
AE003731	AAG22163.1	*"CG4204-PA"*	"[ISS]:transcription elongation factor complex"
AE003732	AAF55809.1	*"CG15693-PA"*	"[ISS]:cytosolic small ribosomal subunit"
AE003763	AAG22173.1	*"CG5502-PA"*	"[ISS]:cytosolic large ribosomal subunit"
AE003792	AAM68401.1	*"CG8900-PB, isoform B"*	"[ISS]:cytosolic small ribosomal subunit"
AE003803	AAF57861.1	*"CG10751-PA"*	"[ISS]:microtubule associated complex"
AE003814	AAF58229.1	*"CG10130-PA"*	"[ISS]:translocon complex"
AE003828	AAF58742.1	*"CG7686-PA"*	
AE003831	AAM71037.1	*"CG30010-PA"*	"[ND]:biological process unknown"
AF181084	AAF16936.1	*"single-strand specific DNA-binding protein"*	
DME271817	CAB72251.1	*"ribosomal protein S17"*	
DMH2AVDG	CAA33555.1	*"histone H2A"*	
DMU66357	AAC47475.1	*"ribosomal protein RpL27a"*	
DROMLCIC	AAA28692.1	*"myosin alkali light chain"*	
DSPTEH2	CAB92783.1	*"nucleolar protein"*	
DVI306692	CAC41629.1	*"ribosomal protein L14"*	

Fungi - GenBank dataset, homology reduced

Locus	Protein Id	"Product" notes
AE017341	AAW40678.1	*"histone binding protein, putative"*
AE017342	AAW41479.1	*"hypothetical protein"*
AE017342	AAW41968.1	*"expressed protein"*
AE017343	AAW42733.1	*"expressed protein"*
AE017344	AAW43030.1	*"adenosylhomocysteinase, putative"*
AE017346	AAW44288.1	*"conserved hypothetical protein"*
AE017350	AAW45899.1	*"60s ribosomal protein l11, putative"*
AE017351	AAW46291.1	*"hypothetical protein"*
AF542530	AAN75619.1	*"RPL22"*
AY426181	AAR84601.1	*"Psa1p"*
BX649606	CAF32004.1	*"60S ribosomal protein l5, putative"*
BX842641	CAE76611.1	*"hypothetical protein"*
BX908809	CAF06043.1	*"putative protein"*
CR380956	CAG60691.1	
CR382124	CAH00415.1	
CR382126	CAG98475.1	
CR382128	CAG83150.1	
CR382130	CAG80890.1	
CR382131	CAG80294.1	
CR382134	CAG85115.1	
CR382138	CAG89397.1	
CR382139	CAG90060.1	
NC12F11	CAC18219.2	*"conserved hypothetical protein"*
NC7F4	CAD70929.1	*"related to secretory pathway protein YSY6"*
NC93G11	CAC28676.2	*"related to calmodulin"*
NCB17B1	CAD70384.1	*"related to ubiquitin fusion degradation protein"*
NCB19A17	CAD70555.1	*"hypothetical protein"*
NCB23B10	CAD70432.1	*"hypothetical protein"*
NCB2O8	CAD01129.1	*"conserved hypothetical protein"*
NCU84904	AAB41886.1	*"V-type ATPase subunit G"*
SC5610	CAA86154.1	
SCD9461	AAB64890.1	*"Rp51bp: ribosomal protein RP51B"*
SCUBC12	CAA67805.1	*"ubiquitin-conjugating enzyme"*
SCYGL030W	CAA96731.1	
SCYGL178W	CAA96889.1	
SCYLR128W	CAA97697.1	
SPAC30D11	CAA91898.1	
SPAC6G9	CAB03609.1	
SPBC211	CAB75413.1	
SPBC29A3	CAA18381.1	
SPBC685	CAB39365.1	
SPBPB2B2	CAC21407.1	
SPCC364	CAA18283.1	
SPDNABRD1	CAA60444.1	*"bromodomain protein"*
YSCRPHOM	AAA35000.1	*"ribosomal protein"*

Magnoliophyta - GenBank dataset, homology reduced

Locus	Protein Id	"Product" notes
AC005395	AAC42252.1	*"hypothetical protein"*
AC006438	AAD41973.1	*"hypothetical protein"*
AC006841	AAD23675.2	*"putative ubiquitin fusion-degradation protein"*
AC007109	AAD25645.1	*"60S ribosomal protein L14"*
AC007196	AAD24820.1	*"putative snRNP splicing factor"*
AC068951	AAM93736.1	*"hypothetical protein"*
AC084295	AAK55474.1	*"putative beta-glucosidase-aggregating factor"*
AC084765	AAP46214.1	*"hypothetical protein"*
AC091627	AAT40513.1	*"hypothetical protein"*
AC092262	AAO37511.1	*"hypothetical protein"*
AC092559	AAO37935.1	*"putative actin-related complex protein"*
AC093713	AAP44665.1	*"putative 40S ribosomal protein S15"*
AC093954	AAS86404.1	*"hypothetical protein"*
AC099040	AAL86501.1	*"putative endonuclease"*
AC099325	AAM18756.1	*"hypothetical protein"*
AC104615	AAN16338.1	*"hypothetical protein"*
AC133005	AAX95860.1	*"hypothetical protein"*
AC135497	AAX94948.1	*"hypothetical protein"*
AC152970	AAW56895.1	*"cell death-related protein"*
AC166741	AAZ41829.1	*"80C09_18"*
AP000399	BAD67661.1	*"hypothetical protein"*
AP001366	BAD81238.1	*"hypothetical protein"*
AP002524	BAD67839.1	*"hypothetical protein"*
AP002864	BAD67972.1	*"hypothetical protein"*
AP003076	BAD86961.1	*"hypothetical protein"*
AP003103	BAD81594.1	*"hypothetical protein"*
AP003104	BAB55737.1	*"hypothetical protein"*
AP003207	BAB64079.1	*"hypothetical protein"*
AP003255	BAD73351.1	*"hypothetical protein"*
AP003263	BAB63622.1	*"putative 40S ribosomal protein S10"*
AP003269	BAD87303.1	*"hypothetical protein"*
AP003316	BAC06253.1	
AP003338	BAD45150.1	*"hypothetical protein"*
AP003346	BAD82125.1	*"hypothetical protein"*
AP003349	BAD82173.1	*"putative ribosomal protein L5"*
AP003508	BAD72466.1	*"hypothetical protein"*
AP003575	BAD53524.1	*"potential autophagy related protein-like"*
AP003617	BAD32869.1	*"hypothetical protein"*
AP003682	BAD53666.1	*"hypothetical protein"*
AP003737	BAD30135.1	*"hypothetical protein"*
AP003747	BAC55602.1	*"unknown protein"*
AP003802	BAC15877.1	*"putative 60S ribosomal protein L44"*
AP003884	BAD01672.1	*"60S ribosomal protein L7A"*
AP004024	BAD27657.1	*"hypothetical protein"*
AP004030	BAD27669.1	*"putative actin related protein 2/3 complex, 2"*

Continues on next page

Locus	Protein Id	"Product" notes
	Continued from previous page	
AP004168	BAD21734.1	*"hypothetical protein"*
AP004300	BAC79847.1	*"putative TGF(transfoming growth factor) bet"*
AP004366	BAD73786.1	*"putative vacuolar-type H(+)-ATPase"*
AP004560	BAC99558.1	*"hypothetical protein"*
AP004650	BAC99594.1	*"hypothetical protein"*
AP004695	BAD12979.1	*"hypothetical protein"*
AP004702	BAD09878.1	*"hypothetical protein"*
AP004772	BAD25374.1	*"hypothetical protein"*
AP004811	BAD33293.1	*"hypothetical protein"*
AP004872	BAD28192.1	*"hypothetical protein"*
AP005003	BAD25654.1	*"hypothetical protein"*
AP005092	BAD33335.1	*"hypothetical protein"*
AP005148	BAD10106.1	*"hypothetical protein"*
AP005191	BAD13074.1	*"hypothetical protein"*
AP005244	BAC16154.1	*"transcription factor-like protein"*
AP005303	BAD22035.1	*"hypothetical protein"*
AP005421	BAD28600.1	*"hypothetical protein"*
AP005479	BAD31504.1	*"hypothetical protein"*
AP005497	BAD38131.1	*"hypothetical protein"*
AP005524	BAD13135.1	*"putative 40S ribosomal protein S25 (RPS25B)"*
AP005546	BAD46229.1	*"putative protein kinase"*
AP005636	BAD28901.1	*"hypothetical protein"*
AP005730	BAD10587.1	*"hypothetical protein"*
AP005896	BAD38405.1	*"hypothetical protein"*
AP006069	BAD17781.1	*"hypothetical protein"*
AP006268	BAD31974.1	*"putative cytoplasmic ribosomal protein L18"*
AP006753	BAD32127.1	*"hypothetical protein"*
AP006860	BAD69384.1	*"hypothetical protein"*
AP008246	BAD89455.1	*"hypothetical protein"*
ATAC009991	AAF01526.1	*"putative 60S ribosomal protein L37a"*
ATCHRIV86	CAB80368.1	*"putative protein"*
ATF13C5	CAA16763.1	*"cadmium-induced protein"*
ATF22O6	CAB43407.1	*"putative ribosomal protein S14"*
ATF24M12	CAB62643.1	*"putative protein"*
ATT16L24	CAB75451.1	*"60S RIBOSOMAL PROTEIN L38-like protein"*
ATT28J14	CAB87265.1	*"ribosomal protein S4"*
ATT30N20	CAB96836.1	*"enhancer of rudimentary"*
ATU78866	AAB68038.1	
OSJN00079	CAE03502.2	
OSJN00089	CAD41812.2	
OSJN00090	CAE03400.3	
OSJN00094	CAE03627.3	
OSJN00096	CAE01864.2	
OSJN00158	CAD40217.2	
OSJN00163	CAD40354.1	
U90439	AAF18683.2	*"putative guanylate kinase"*
